# Primary and secondary thyroid hormone transporters

**DOI:** 10.1186/1756-6614-4-S1-S7

**Published:** 2011-08-03

**Authors:** Anita Kinne, Ralf Schülein, Gerd Krause

**Affiliations:** 1 Leibniz-Institut für Molekulare Pharmakologie (FMP), Robert-Roessle-Str. 10, 13125 Berlin, Germany

## Abstract

Thyroid hormones (TH) are essential for the development of the human brain, growth and cellular metabolism. Investigation of TH transporters became one of the emerging fields in thyroid research after the discovery of inactivating mutations in the Monocarboxylate transporter 8 (MCT8), which was found to be highly specific for TH transport. However, additional transmembrane transporters are also very important for TH uptake and efflux in different cell types. They transport TH as secondary substrates and include the aromatic amino acid transporting MCT10, the organic anion transporting polypeptides (e.g. OATP1C1, OATP1A2, OPTP1A4) and the large neutral amino acid transporters (LAT1 and LAT2). These TH transporters characteristically possess 12 transmembrane spanners but due to the strong differing sequences between the three transporter families we assume an identical conformation is not very likely. In contrast to the others, the LAT family members form a heterodimer with the escort protein 4F2hc/CD98. A comparison of sequence proportions, locations and types of functional sensitive features for TH transport discovered by mutations, revealed that transport sensitive charged residues occur as conserved amino acids only within each family of the transporter types but not in all putative TH transporters. Based on the lack of highly conserved sensitive charged residues throughout the three transporter families as a common counterpart for the amino acid moiety of the substrates, we conclude that the molecular transport mechanism is likely organized either a) by different molecular determinants in the divergent transporter types or b) the counterparts for the substrates` amino acid moiety at the transporter are not any charged side chains but other proton acceptors or donators. However, positions of transport sensitive residues coincide at transmembrane helix 8 in the TH transporter MCT8, OATP1C1 and another amino acid transporter, the L-cystine and L-glutamate exchanger xCT, which is highly homologous to LAT1 and LAT2. Here we review the data available and compare similarities and differences between these primary and secondary TH transporters regarding sequences, topology, potential structures, trafficking to the plasma membrane, molecular features and locations of transport sensitive functionalities. Thereby, we focus on TH transporters occurring in the blood-brain barrier.

## Introduction

Investigation of thyroid hormone (TH) transporters has become one of the emerging fields in thyroid research during the last few years. Molecular studies of TH transporters were enforced after the discovery of inactivating mutations in the TH transporter MCT8 (Monocarboxylate transporter 8). These mutations cause the Allan-Herndon-Dudley syndrome (AHDS) [1], which is an X-linked mental retardation. The affected patients show normal TSH (Thyroid-stimulating hormone, thyrotropin) but elevated T_3_ (3,3',5-triiodo-L-thyronine) and decreased T_4_ (3,3',5,5`-tetraiodo-L-thyronine) serum levels [2-6].

TH are essential for the development of the human brain, growth and cellular metabolism. A dysfunction in the availability of TH during early embryonic development leads to neurological deficiency [7]. In the brain, TH are important e.g. for the timely migration of neurons, formation of synaptic contacts and myelination [8]. Neurons are the major target cells for T_3_ during brain development. According to current concepts, the prohormone T_4_ enters the neighbouring astrocyte and deiodinase 2 converts T_4_ to the active form T_3_ which is then transported into neurons by MCT8 [9].

An impaired uptake of T_3_ in MCT8-expressing central neurons could explain the neurological deficits found in AHDS patients [1,3]. Surprisingly, the corresponding Mct8-deficient mice show the endocrine changes only, but not the neurological phenotype, observed in affected humans. Symptoms of hyperthyroidism in the peripheral tissues in combination with symptoms of hypothyroidism in the central nervous system were shown in mice. The brain uptake of exogenous T_3_ is also markedly reduced, whereas the uptake of T_4_ shows 50% transport activity in comparison to the wild type mice [10-12].

On the basis of these data it was assumed that additional TH transporters (“secondary TH transporters”) or differences in their expression patterns and substrate specificity can compensate the loss of Mct8 in mice [13]. Finally, it became obvious that secondary TH transporters are also very important for TH uptake and efflux in different cells types. Whereas the primary TH transporter MCT8 is currently known to be highly specific for TH only [14], the secondary TH transporters are also able to transport different kinds of amino acids and comprise the aromatic amino acid transporting MCT10, the organic anion transporting polypeptide transporters (e.g. OATP1C1, OATP1A2, OPTP1A4), and the large neutral amino acid transporters (LAT1 and LAT2).

Another amino acid transporter, the sodium independent exchanger of L-cystine and L-glutamate (xCT), which can even transport negatively charged amino acids, is also considered here, since xCT shows close similarities to LAT transporters regarding molecular features and plasma membrane trafficking mechanism.

Therefore, we here review the data available on similarities and differences between the primary and secondary TH transporters regarding sequence, trafficking to the plasma membrane, molecular features, predicted membrane topologies, potential structures and functionalities. Thereby, our focus here is on TH transporters occurring in the blood-brain barrier (BBB).

## Expression patterns of primary and secondary TH transporters

It was shown in the brain that MCT8 expression in neurons is essential for neuronal uptake of T_3_[15]. The reduced brain uptake of T_3_ and T_4_ in Mct8-deficient mice suggests that MCT8 transports TH across the BBB. *Roberts et al.* could show an expression of MCT8 in cerebral microvessels and demonstrated the expression of MCT8 at the BBB in the human, mouse, and rat brain [13]. These expression profiles support the suggestion that MCT8 does not only play a major role in TH action in the brain in humans and rodents but also in the uptake of TH in the brain across the BBB in humans. MCT8 is also expressed in the thyroid, liver, testis, and in the skeletal muscle (table 1). Another member of the MCT family which transports TH, MCT10, shows overlapping expression patterns with MCT8, except that MCT10 has not been detected in the thyroid, brain and testis (table 1).

**Table 1:** Tissue distribution of TH transporters from the MCT (MCT8 and MCT10), OATP (OATP1C1, OATP1A2, OATP1A4), and LAT (LAT1 and LAT2) family and of the xCT transporter and the escort protein 4F2hc.

Gene	Protein	Localization	References
SLC16A2	MCT8	liver, kidney, brain, heart, skeletal muscle, placenta, thyroid, testis	[14,15,33,64-68]
SLC16A10	MCT10	intestine, kidney, liver, skeletal muscle, heart, placenta, pancreas	[67-69]

SLCO1C1	OATP1C1	brain, testis, cochlea	[38,39]
SLCO1A2	OATP1A2	brain, liver, kidney, intestine	[70-72]
SLCO1A4	OATP1A4	liver, brain, testis, ovaries, retina	[23,70,73]
SLC7A5	LAT1	multiple (tumours, brain, spleen, placenta, testis, colon, kidney, intestine, stomach, ovary, thymus, not liver)	[74-81]
SLC7A8	LAT2	kidney, placenta, brain, intestine, testis, ovary, liver, heart, skeletal muscle, lung, stomach	[74,79-83]

SLC7A11	xCT	brain, kidney, activated macrophages, duodenum	[20,84,85]
			
**Gene**	**Escort protein**	**Localization**	**Reference**

SLC3A2	4F2hc (CD98)	multiple (tumours, brain, intestine, kidney, liver, skeletal muscle, ovary, placenta, testis…)	[74]

OATP1C1 like MCT8 is expressed at the BBB in mouse and rat, but to a much lesser extent at the human BBB [13]. The strong expression of OATP1C1 in the rodent but less so in the human BBB could explain why Mct8-deficient mice do not show the neurological changes found in AHDS patients. The OATP family members OATP1A2 and OATP1A4 are also expressed in the brain and show transport of TH (table 1) [16].

**Figure 1:**
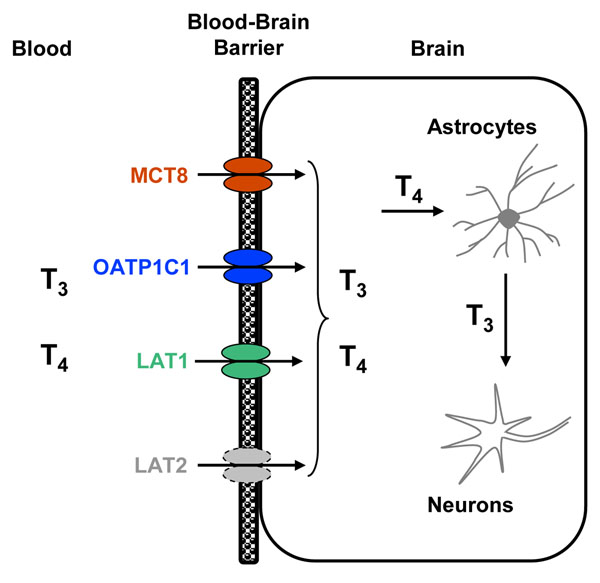
**Illustration of the TH transporters expressed at the blood-brain barrier (BBB).** The TH transporters MCT8, OATP1C1, LAT1, and LAT2 are expressed at the BBB, whereby LAT2 shows lower expression levels than the LAT1. The prohormone T_4_ enters the astrocyte and is converted to the active form T_3_ by an outer ring deiodination. According to current concepts, T_3_ enters the neurons by the TH transporter MCT8.

The TH transporters LAT1 and LAT2 are expressed in various tissues, e.g. in luminal and abluminal membranes of brain capillary endothelial cells, placenta, and intestine, whereas LAT1 also shows high expression levels in tumour cells (table 1). Both transporters are expressed at the BBB, whereby LAT1 expression is higher than LAT2 [17] (figure 1). LAT1 and LAT2 form a heterodimer with the escort protein, 4F2hc (4F2 heavy chain, CD98), which is ubiquitously expressed, e.g. in tumours, brain, kidney, intestine, and placenta (table 1) [18,19].

The amino acid transporter xCT is also expressed in the brain (table 1) [20,21] and it has been suggested that xCT is up-regulated in glial cells upon the oxidative stress and plays an essential role to protect neurons against oxidative stress [22].

## Topology and structures of TH transporters

The TH transporters considered here show a common topology by comprising 12 transmembrane helices (TMHs) while the N- and C-terminal tails are located intracellularly, like a vast number of other known transporters of the major facilitator superfamily (MFS) (see transporter database http://www.tcdb.org/superfamily.php). The MFS is an evolutionary diverse superfamily that includes over 10,000 sequenced members which catalyze uniport, symport and antiport transport mechanisms.

MCT8 and MCT10 belong to the MFS subgroup of Monocarboxylate transporter family. Since the monocarboxylate transport for the members MCT1-4 is proton linked, these MCT transporters are not further considered here.

The OATP family members Oatp1a4 and Oatp1a5 were the first cloned TH transporters [23]. Although the OATP family also belongs to the MFS in terms of sequence similarity it is more distant from MCT8 and MCT10.

LAT1 and LAT2 are even more distant to MCT8. They are members of the Amino Acid-Polyamine-Organocation (APC) superfamily and transport sodium-independent large neutral amino acids such as phenylalanine, tyrosine, leucine, arginine and tryptophan from extracellular fluids into the cell, when associated with SLC3A2/4F2hc. The glutamate transporting xCT also belongs to the APC family and shows high similarities to the LATs on the molecular level.

There is a lack of detailed experimental structural information for all TH transmembrane transporters. Therefore, the crystal structure of the Glycerol-3-phosphate transporter (GlpT, PDB code 1PW4 [24]), another member of the MSF superfamily, has been used as a structural template for homology models for MCT1 [25], MCT8 [26], and OATP1C1 [27]. Although it has been suggested that the substrate translocation at GlpT is associated with conformational changes by an alternating access mechanism with a rocker-switch type of movement of the N- and C-terminal domains [23,25], details for the particular TH transporters are not known yet.

Among the numerous X-ray structures of bacterial transporters the high-resolution three-dimensional structures of another MFS type, the lactose H^+^ symporter (LacY) [28,29], also shows the closest structural homology to GlpT. The crystal structure of an APC family transporter, the proton-coupled broad-specificity amino acid transporter apo-ApcT [30], shows the 12 TMHs in an inward-facing apo state. It shows a structural similarity to the 12 TMHs within the crystal structure of a bacterial homologue for neurotransmitter transporters, the sodium-dependent leucine transporter (LeuT) from *Aquifex aeolicus*[31]*.* The ion independent GlpT structure possesses a single binding site for the substrate. The other available structures from MSF and APC families are from proton- or ion-coupled transporters and contain additional binding sites for the symported protons or ions.

## Substrate spectra of TH transporters show a broad range

So far cellular uptake of TH has been shown for the monocarboxylate transporters MCT8 and MCT10, the organic anion transporting polypeptides, e.g. OATP1C1, OATP1A2, and OATP1A4, and the heterodimeric amino acid transporters LAT1 and LAT2 [16].

MCT8 was identified as the first specific transporter of T_4_ and T_3_ and was found to transport their inactive TH metabolites such as rT_3_ (3,3',5`-triiodo-L-thyronine) and 3,3`-T_2_ (3,3`-diiodo-L-thyronine) [14]. Thereby, the transport of T_3_ is higher than of T_4_ or rT_3_, respectively (T_3_ > T_4_ > rT_3_ ~ 3,3`-T_2_) (table 2) [14]. In order to be transported by human MCT8, substrates require at least one iodine atom per aromatic ring preferably at their 3 and 3`position, respectively [26]. Neither thyronamines (except 3-iodothyronamine [32]), decarboxylated metabolites of iodothyronines, nor TH derivatives lacking both chiral center and amino group, are substrates for MCT8 [26,32]. MCT10 was first identified as a T-type transporter for aromatic amino acids [33] and later *Friesema et al.* showed that MCT10 is at least as active for TH transport as MCT8 [34].

**Table 2:** Uptake of iodothyronines by TH transporters expressed at the blood-brain barrier. The levels of TH transport are indicated as follows: +++ high uptake rate, ++ modest uptake rate, and + low uptake rate; n.d., not determined.

Protein	Iodothyronines				Species	References
	**3,3`-T**_**2**_	**T**_**3**_	**rT**_**3**_	**T**_**4**_		
MCT8	+/+	+++/+++	+/++	++/++	human/rat	[14,86]
OATP1C1	n.d.	+	+++	+++	human, rat, mouse	[37-39]
LAT1	+++	++	++	+	human	[43]
LAT2	+++	++	++	+	mouse	[43]

The TH transporting OATP family members show a broader substrate spectrum, e.g. in addition to TH they transport bile acids, steroid hormones [35] and drugs [36]. Functional transport analysis of OATP1C1 revealed that T_4_ and rT_3_ are high affinity substrates of OATP1C1, whereas the specific uptake of T_3_ is about 5-fold less than that of T_4_ and rT_3_ (T_4_ ~ rT_3_ > T_3_) [37-39] (table 2). It has been suggested that OATP1C1 is primarily responsible for T_4_ uptake from the blood into the brain across the BBB where it is locally converted to the active T_3_, which is in turn transported into neurons by MCT8 [40].

LAT1 and LAT2 transport large neutral amino acids and amino acid related compounds, whereas LAT2 also transports small amino acids [41,42]. Functional analysis of LAT1 could demonstrate the iodothyronine uptake, which decreased in the order 3,3`-T_2_ > rT_3_ ~ T_3_ > T_4_. Smaller increments in iodothyronine uptake were noted in transport mediated by LAT2 (table 2) [43].

The amino acid transporter xCT is responsible for the cystine transport through the plasma membrane [44,45]. It mediates an amino acid exchange and prefers cystine and glutamate as its substrates [46].

## Similarities and Differences of TH transporters on Molecular Level

### Sequences

**Figure 2:**
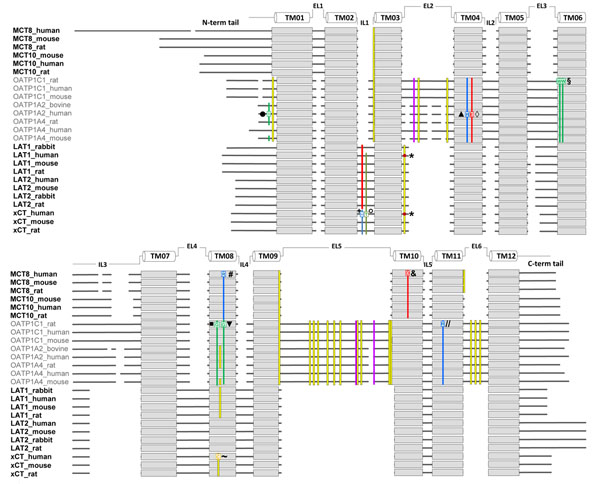
**Comparison of sequence proportions and functional sensitive features of primary and secondary TH transporters.** Sequence scheme of TH transporters expressed at the blood-brain barrier (BBB) is aligned according to the sequences of common 12 transmembrane spanners (pale grey boxes). Sequence lengths, proportions and known sensitive features of transport differ between the three TH transporter families (MCT, OATP, and LAT). The xCT sequences are very homologous to LATs and are therefore added. The human MCT8 possesses a very large N-terminal tail at the intracellular portion. Intra- and extracellularly conserved cysteines are marked in yellow bars. N-glycosylation sites of the OATP family are conserved at large extracellular loops EL2 and EL5 (magenta bars). The proven disulfide bridge formed by the LAT and xCT family to the escort protein 4F2hc are conserved in the EL2 (C165-LAT1_human [49] or C158-xCT_human [50] marked by red dot * at yellow bar). Sensitive positions for TH transport identified by mutations are highlighted: MCT8_human: R445A(# blue bar, TMH8 [26]) and D498A (& red bar, TMH10 [26]), the sensitive arginine and aspartate are conserved at the MCT8 and MCT10 group; Oatp1c1_rat: W277A and W278A (§ green bars, TMH6 [27]), G400A and G410A (■ and ▼green bars, TMH8 [30]), R601A (// blue bar, TMH11 [27]); OATP1A2_human: I13T (◊ N-terminal tail [63]), R168C (▲ blue bar, TMH4 [58]), E172D (◊ red bar, TMH4 [63]). The isoleucine at position 13 (green bar, N-terminal tail) is only conserved at the OATP1A2 group, while the sensitive tryptophan (green bars), arginines (blue bars) and glutamate (red bar) are conserved within the OATP family. At the human xCT may lie close to the substrate binding site H110 (+ blue bar [21]) and T112 (○ green bar [21]) in IL1 and the C327 (~ yellow bar [62]) at TMH8. The accession numbers of these proteins are NM_006517.3 (MCT8_human), NM_009197.2 (MCT8_mouse), NM_147216.1 (MCT8_rat), NM_018593.4 (MCT10_human), NM_001114332.1 (MCT10_mouse), Q91Y77 (MCT10_rat), NM_017435.4 (OATP1C1_human), NM_021471.2 (OATP1C1_mouse), NM_053441.1 (OATP1C1_rat), NM_134431.3 (OATP1A2_human), NM_174654.2 (OATP1A2_bovine), AF205071.1 (OATP1A4_human), NM_030687.1 (OATP1A4_mouse), NM_131906.1 (OATP1A4_rat), BC039692.2 (LAT1_human), BC026131.1 (LAT1_mouse), CH473972.1 (LAT1_rat), NM_001082120.1 (LAT1_rabbit), BC052250.1 (LAT2_human), BC059004.1 (LAT2_mouse), NM_053442.1 (LAT2_rat), NM_001082682.1 (LAT2_rabbit), NM_014331.3 (xCT_human), NM_011990.2 (xCT_mouse), NM_001107673.2 (xCT_rat).

A comparison of the different sequence proportions and features among the discussed TH transporters is shown in figure 2. Sequence lengths, proportions and known sensitive features of transport differ between the three TH transporter families (MCT, OATP, and LAT). Due to the very high sequence similarity to the LAT sequences, we also considered the xCT sequences here.

Sequence alignment reveals that the MCT8 and especially the human MCT8 contain a very large N-terminal tail, for which the function is still unknown [47]. Both TH transporting MCT family members, MCT8 and its close homolog MCT10, do not contain extracellular glycosylation sites [34].

Other secondary TH transporters such as those from the OATP family are less similar. They show larger extracellular loops (EL) with numerous conserved cysteines in EL2, EL5 and EL6 (yellow bars in figure 2) that are probably disulfide bridged. Moreover, chances are that several conserved glycosylation sites in EL2 and EL5 are responsible for appropriate traffic of this transporter type towards the cell membrane (magenta bars in figure 2).

The transporters that belong to the APC superfamily, such as LAT1/2 and xCT, are even more evolutionary distant from MCT8. Like the other TH transporters they possess 12 transmembrane α-helical spanners but for suitable membrane expression they need the one helix transmembrane spanner escort protein 4F2hc [48-50]. This transporter type lacks any glycosylation site since the glycosylation sites are only comprised at the large extracellular portion of 4F2hc. Thus, both transporter types (LAT and xCT) are in fact tightly associated by a 13th TMH that is provided by the escort protein 4F2hc. This odd number of transmembrane segments might be one reason why the potential intracellular loop 1 (IL1) between TMH2 and TMH3 is organized as a re-entrant loop in the xCT. In a detailed experimental study elucidating the topology of xCT [21] it was shown that histidin 110 in IL1 (marked + in figure 2) and threonin 112 (marked ° in figure 2) are spatially located in human xCT in such a way that they are accessible from the extracellular side. At the xCT, a part of IL1 is interposed between the transmembrane segments in such a manner that the two residues are accessible from the extracellular side.

The number and properties of residues at IL1 are highly conserved among xCT and LAT1/2. Moreover, at the corresponding amino acid positions, where extracellular accessibility was shown in xCT (H110 and T112), the hydrophilic residues aspartate and serine appear in LAT1 and LAT2 respectively (red and grey bars at IL1 in figure 2). Therefore it is feasible that the IL1 may also functions as a re-entrant loop in LAT1 and LAT2.

### Escort proteins

Not only differences in the transport of TH within TH transporters are known, but the trafficking mechanisms of these transporter proteins towards the plasma membrane differ also. Trafficking of integral membrane proteins to the plasma membrane is mediated by the secretory pathway. At the beginning, proteins are integrated into the membrane of the endoplasmatic reticulum and are then delivered in the membrane of vesicles through the individual compartments of the Golgi apparatus to the plasma membrane. MCT8, MCT10, LAT1, and LAT2 are non-glycosylated proteins, whereas the OATPs possess potential glycosylation sites [51] (figure 2). Normally, glycosylations are essential for trafficking whereby the trafficking mechanisms for the two TH transporters from the MCT and the LAT families are organized differently. In the case of MCT8 and MCT10 it is speculated that the large N-terminal tail might contain features that facilitate trafficking towards the membrane. Other transporters of the MCT family such as MCT1 and MCT4, which do not transport TH but other substrates e.g. lactate, pyruvate and ketone bodies [52], require an association with the escort protein CD147 for efficient cell surface expression [53] instead. In contrast to the TH transporting MCT family members, the two LAT transporters also need an escort protein for efficient cell surface translocation, the 4F2hc [18,19].

The two escort proteins 4F2hc and CD147 share a common topology. The amino-terminus of 4F2hc is located intracellularly, whereas the very large extracellular carboxy-terminus [48] contains four potential glycosylation sites [54]. Thus, 4F2hc is a multifunctional type II membrane glycoprotein [55]. In the heterodimer complex between LAT1/2 and 4F2hc the 12 helix transport protein is called the light chain and the glycosylated escort protein 4F2hc is called the heavy chain. The heavy chain can form heterodimers with six different non-glycosylated light chains like LAT1, LAT2, y+LAT1, y+LAT2, asc1, and xCT [48].

Both subunits of the heterodimer between the L-type amino acid transporters build a covalent bond via a disulfide bridge through the cysteine residue 109 of the extracellular region of human 4F2hc and the cysteine residue 165 in the second extracellular loop localized between the TMH3 and TMH4 of rat Lat1 (red dot and * in figure 2) [48,49]. This cysteine residue is conserved in all sequences of LAT1, LAT2 and also in all xCT sequences in EL2 (yellow bar in figure 2).

Although these two subunits of the heterodimeric transporters are joined by the highly conserved disulfide bridge, site-directed mutagenesis of these two cysteines on either LAT1 or 4F2hc does not inhibit amino acid transport [56].

Moreover, according to the high sequence homology, it could be shown that the corresponding cysteine in human xCT (cysteine 158, red dot and * in figure 2) [57] also forms a disulfide bridge to 4F2hc.

## Functional data for amino acids potentially involved in substrate interaction and functional transport

Different functional studies of TH transporters could identify amino acid residues potentially involved in substrate interaction. To gain insights into structure-function relationship in TH transport, we designed the first structural model for human MCT8. Thereby, the conserved and charged amino acids R445 at TMH8 (marked # in figure 2) and D498 at TMH10 (marked & in figure 2) have been identified as being involved in substrate interaction. An abrogated T_3_ transport by the alanine mutants R445A and D498A supported their predicted role in substrate recognition, although both mutants were exposed to the cell surface [26].

The sensitive arginine (blue bar) and aspartate (red bar) are conserved at the MCT8 and MCT10 group in TMH8 and TMH10, respectively (figure 2). The MCT8 model allows to identify transport characteristics of TH and to rationalize potential interactions of amino acids including those mutated in patients with AHDS.

In the high-affinity T_4_ transporter Oatp1c1 *Westholm et al.* identified amino acid residues to be critical for T_4_ transport and demonstrated the presence of high and low affinity binding sites [27]. In their study they mapped rat Oatp1c1 substrate interacting sites. W277 and W278 (§ in figure 2) were shown to play a major role in T_4_ transport with direct binding effects on one binding site.

Oatp1c1 mutations that reach the cell membrane but affect the transport function such as G400A/V and G410A/V at TMH8 (marked ■ and ▼ in figure 2) were proposedly involved in transport kinetics. Their alanine and also valine mutants displayed wild type-like uptake activity but exhibit diminished T_4_ transport at high substrate concentrations. The authors suggested that a substrate binding site might collapse or is turned to inability to convert between input and output states. Based on mutants of the conserved arginine R601 (marked // in figure 2) at TMH11 of rat Oatp1c1 the authors suggested that this arginine may serve within OATP members as a countercharge for anionic binding to OATP1C1 [27]. Genetic variations by Single nucleotide polymorphisms (SNPs) have been detected to be involved in disturbed substrate transport like methotrexate transport at human OATP1A2 [58] such as R168C at TMH4 [58] (marked ▲ in figure 2). Recently it has been proven that some of these SNPs such as I13T at the intracellular N-terminal tail (marked ● in figure 2), and E172D at TMH4 (marked ◊ in figure 2), also effect the T_3_ and T_4_ transport [59].

Our sequence comparison scheme showed that the isoleucine (marked ● in figure 2) at amino acid position 13 at the N-terminal tail is only conserved at the OATP1A2 group (green bar in figure 2), while the other identified TH sensitive residues such as arginine (blue bars in figure 2) and glutamate (red bar in figure 2) located at TMH4, the two tryptophans (green bars in figure 2) at TMH6, the two glycines (green bars) at TMH8 and the arginine (blue bar) in TMH11 are conserved within the OATP family.

Thereby it is noticeable that the TH transport sensitive position glycine 410 (marked &#9679 in figure 2) of rat Oatp1c1 in TMH8 corresponds to the position of the transport sensitive arginine 445 (marked # in figure 2) in TMH8 at human MCT8.

*Boado et al.* identified amino acids in the L-type amino acid transporter LAT1 from rabbit, which are potentially involved in trafficking mechanisms to the plasma membrane. They showed that the cysteine residue 439 (corresponding to C443 in human LAT1) plays a significant role at TMH11 in either folding or insertion of the transporter protein in the plasma membrane [56]. Mutagenic analysis of the amino acids G219 and W234 in rabbit LAT1, which differ in relation to the human or rat LAT1, demonstrated marked changes in the affinity and capacity of LAT1 [60]. In order to identify domains involved in recognition of the light chains, LAT1, LAT2, and y+LAT2 by 4F2hc were investigated by *Bröer et al..* They suggested that extracellular domains of the 4F2hc are mainly responsible for recognition of light chains other than LAT1 and that the extracellular domain ensures proper translocation to the plasma membrane [61]. Functional data for xCT demonstrated that H110 located in IL1 which here also represents the re-entrant loop (marked + in figure 2) may lie close to the substrate binding/permeation pathway of xCT [21]. Studies of the cysteine residue 327 at TMH8 of xCT (marked ~ in figure 2) indicated that it is also located close to the substrate binding site of xCT [62]. This transport sensitive cysteine at TMH8 is conserved in xCT and LAT1 transporters but not in LAT2. In addition, it occurs sporadically at the OATP group.

Residues that are sensitive for TH transport have been found for MCT8 on TMH8 and TMH10 and priorily on TMH4, TMH6, TMH8 and TMH11 for Oatplcl. Until now, no TH transport sensitive residues have been reported for LAT1 and LAT2. For the highly homologous xCT relevant residues for substrate transport have, however, been identified at the re-entrant loop IL1 and on TMH8. When comparing the transport sensitive features by mapping them on a sequence alignment scheme (figure 2), it becomes clear that the identified residues are almost only conserved within one particular TH transporter family. None of the sensitive residues are highly conserved across all three considered families. However, the location of a TH sensitive glycine at TMH8 of the Oatp1c1 and a substrate sensitive cysteine at TMH8 of the xCT corresponds to a similar region, where in TMH8 of the MCT8 a TH transport sensitive arginine was found (figure 2).

## Conclusions

A comparison of TH transporter sequences reveals that with the exception of MCT10 secondary TH transporters belonging to different transporter type families are more divergent to the primary TH transporter MCT8. All different endogenous substrates that are transported by these diverse transporters contain at least one common molecular feature, namely the amino and carboxylate functional group of amino acids. A comparison of locations and types of the identified sensitive TH transporting residues revealed that the known positive or negative charged residues occur as conserved amino acids within each of the transporter types, but not over all TH transporters. Due to the fact that highly diverse amino acid properties (arginine and glycine respectively) have been identified as sensitive for TH transport at a corresponding position of TMH8 for MCT8 and Oatp1c1, we presume that molecular details of the translocation mechanisms are varying between MCT8 and Oatp1c1. From the divergent sequence features among the three secondary TH transporter types and the lack of any highly conserved sensitive positively or negatively charged residues as a common counterpart for the amino acid moiety of the substrates, we conclude that the molecular transport mechanisms are likely organized either a) by different molecular determinants in the divergent transporter types or b) the counterpart for amino acid moiety of the substrates at the transporter are not charged side chains but other proton acceptors or donators. Moreover, even though the TH transporters share the common 12 transmembrane spanners, an identical conformation for all TH transporters is not very likely, particularly at least for the LAT family exhibiting an associated 13th transmembrane helix that is provided by the escort protein. A distinct conformation of the two LATs is also supported by the findings for the homologous xCT suggesting a re-entrant function of the IL1. On the other hand the coincidence of sensitive positions for substrate transport at TMH8 (although by divergent residues) in the different TH transporters MCT8, OATP1C1 and even in xCT could at least be a hint for shared molecular transport events executed at a comparable interior site on TMH8 of the transporters considered here.

Therefore, a detailed knowledge of molecular mechanisms of TH translocation through the membrane transporting proteins by investigation of structure-function relationships between different substrates and their targets is of vital importance to understand the molecular reasons for defects in TH transport. In this respect, future investigations of the molecular mechanisms for different substrate transport in the divergent TH transporters are of great importance to clarify the structural-functional properties of TH transport.

## Competing interests

The authors declare that they have no competing interests.

## Authors' contributions

AK wrote the draft, analyzed the data, prepared the figures, data evaluation and discussion; RS wrote the draft, data discussion, project coordination; GK wrote the draft, analyzed the data, data evaluation and discussion, project coordination.

## Abbreviations

AHDS: Allan-Herndon-Dudley syndrome; APC superfamily: Amino Acid-Polyamine-Organocation superfamily; asc1: asc-type amino acid transporter 1; BBB: blood-brain barrier; LAT: L-type amino acid transporter; MCT: Monocarboxylate transporter; MFS: major facilitator superfamily; OATP: organic anion transporting polypeptide; SNPs: Single nucleotide polymorphisms; TMH: transmembrane helix; rT_3_: 3,3',5`-triiodo-L-thyronine; 3,3`-T_2_: 3,3`-diiodo-L-thyronine; T_3_: 3,3',5-triiodo-L-thyronine; T_4_: 3,3’,5,5’-tetraiodo-L-thyronine; TH: thyroid hormone; TSH: Thyroid-stimulating hormone, thyrotropin; 4F2hc: 4F2 heavy chain, CD98; xCT: cystine/glutamate exchanger; y+LAT1: y(+)L-type amino acid transporter 1.
